# A Guide on Deep Learning for Complex Trait Genomic Prediction

**DOI:** 10.3390/genes10070553

**Published:** 2019-07-20

**Authors:** Miguel Pérez-Enciso, Laura M. Zingaretti

**Affiliations:** 1 Catalan Institution for Research and Advanced Studies (ICREA), Passeig de Lluís Companys 23, 08010 Barcelona, Spain; 2 Centre for Research in Agricultural Genomics (CRAG), CSIC-IRTA-UAB-UB, 08193 Bellaterra, Spain

**Keywords:** deep learning, genomic prediction, machine learning

## Abstract

Deep learning (DL) has emerged as a powerful tool to make accurate predictions from complex data such as image, text, or video. However, its ability to predict phenotypic values from molecular data is less well studied. Here, we describe the theoretical foundations of DL and provide a generic code that can be easily modified to suit specific needs. DL comprises a wide variety of algorithms which depend on numerous hyperparameters. Careful optimization of hyperparameter values is critical to avoid overfitting. Among the DL architectures currently tested in genomic prediction, convolutional neural networks (CNNs) seem more promising than multilayer perceptrons (MLPs). A limitation of DL is in interpreting the results. This may not be relevant for genomic prediction in plant or animal breeding but can be critical when deciding the genetic risk to a disease. Although DL technologies are not “plug-and-play”, they are easily implemented using Keras and TensorFlow public software. To illustrate the principles described here, we implemented a Keras-based code in GitHub.

## 1. Introduction

Most agriculturally important traits (e.g., average yield or drought tolerance in plants and litter size or milk production in animals), as well as susceptibility to numerous diseases, exhibit a complex genetic architecture. This makes it very difficult to identify individual causal loci. In practice, then, the prediction of breeding values or disease risk relies on statistical techniques that typically assume the well-known infinitesimal model [1]. With the availability of high throughput genotyping technology, genomic prediction has become the standard method in breeding schemes. Genomic prediction is a procedure whereby unobserved complex traits of individuals are predicted from their observed genomic information (typically single nucleotide polymorphism, SNPs, or whole genome sequence data). This idea was originally proposed to improve animal breeding programs by Meuwissen et al. [2]. Since then, numerous methodological and practical developments have vastly increased the catalog of predictive methods [3]. Genomic prediction was first implemented in animal species with long generation intervals such as dairy cattle. Other animal and plant breeding schemes are slowly but steadily catching up. Genomic prediction should be particularly useful in tree species given their long generation intervals [4]. In humans, these techniques have been proposed to predict future predisposition to genetically inherited diseases (genetic risk) or to analyze complex quantitative traits such as height [5,6].

Traditionally, genomic prediction has been based on genotyping arrays. More recently, with the advent of next generation sequencing (NGS) technologies, using a complete sequence for prediction has become feasible, or at least possible. In principle, the NGS data offer numerous advantages over using solely SNP arrays, i.e., the causal mutations should be in the data and disequilibrium between causal SNPs and traits would not decrease with time, avoiding the need to recalibrate the model every few generations [7]. In practice, however, both simulation and empirical studies have not confirmed a significant advantage of sequence over high density SNP arrays [8,9].

Irrespective of the type of molecular data, numerous techniques have been proposed for genomic prediction. Among these, “deep learning” (DL) algorithms have recently attracted interest, although their performance in the prediction of complex traits is not well studied. Deep learning (DL) algorithms are generic and highly flexible tools that have led to success in diverse areas (e.g., analyses of image, video, voice, text, and protein folding). DL algorithms have already been applied to a wide variety of genomic problems such as somatic variant calling [10] and prediction of clinical impact of mutations [11] or transcription patterns [12]. See also reviews on this topic in [13,14].

Single-layer neural networks have been employed in animal and plant breeding, e.g., [15,16], see also the review in [17]. In general, these shallow neural networks have been found to be highly prone to overfitting, although they were sometimes competitive with penalized linear methods. The purpose of this work is to focus on DL specific applications to genomic prediction, since there are still few results on this topic, as well as provide a practical guide on how to use DL in this context.

This work is organized as follows: First, we provide a basic outline of genomic prediction theory and current limitations. Second, we describe the theoretical concepts behind DL, including main DL architectures, optimization, and modeling strategies. These parts can be skipped by the less mathematically motivated reader but are helpful to understand implementation options. Third, we show how, despite all complexities, running DL algorithms is extremely easy, and we provide detailed, easy to modify code in https://github.com/miguelperezenciso/DLpipeline (Supplementary File). Fourth, we discuss some issues of particular interest in the area, such as DL interpretability, and we review current results of applying DL for genomic prediction. Finally, we provide practical recommendations.

## 2. Genomic Prediction Principles and Current Limitations

In a genomic prediction problem, the following model is usually defined by the following equation:

(1) y=Xβ+Zm+ε

where, *y* is a vector of observed phenotypes, β contains the fixed effects, X represents its incidence matrix, m is a vector of (random) marker effects, whereas, Z is its incidence matrix (i.e., genotypes). Finally, ε is a residual term. In Equation (1), *y* is either continuous or categorical. In the latter case, a probit or logistic transformation is used [18]. Since the number of markers usually exceeds the number of records, especially in the case of sequence or high-density SNP arrays, Equation (1) requires some sort of penalization or “constraint” on the solutions. In fact, the main difference between genomic prediction methods is the type of penalty applied to marker information [3].

Standard genomic prediction models have been developed from a frequentist (e.g., genomic best linear unbiased prediction GBLUP, LASSO [19,20]) or Bayesian (Bayes A, B [2,18]) points of view. Frequentist methods require strong assumptions, for example, on data distributions, since they focus on inference, i.e., hypothesis testing and parameter estimation. Bayesian methods allow us to obtain the full posterior distribution of parameters, although they require priors’ specification. In contrast, methods developed for the sole purpose of prediction, such as DL, can be less restrictive because their only aim is to predict new data as accurately as possible.

Among the most prominent advantages of DL for the purposes of genomic prediction is their ability to learn without model assumptions. This is relevant as there is no need to specify, e.g., whether the phenotype shows dominance or epistasis. Moreover, DL models nonlinear relationships since DL admits numerous nonlinear activation functions. Provided enough data are available, it should be possible to find the best DL architecture, that is able to learn by itself, irrespective of the underlying genetic architecture.

## 3. Deep Learning Principles

A generic DL architecture is made up of a combination of several layers of “neurons”. The concept of a neural network, which is the core of DL, was proposed in the 1950s with the well-known Rosenblatt “perceptron”, inspired on brain function [21]. The DL revival, during the last decade, was due to the discovery of efficient algorithms that are able to estimate parameters in complex networks made up of several neuron layers (e.g., backpropagation [22]) and to the fact that these methods clearly outperform current algorithms in several automatic recognition tasks such as image analysis [23]. Deep learning is an area with numerous specific jargon terms, therefore, to facilitate understanding for the unexperienced user, some of the most relevant concepts are defined in Table 1.

### 3.1. Main Deep Learning Architectures

Although all DL methods share the common principle of using stacked layers of neurons, they actually comprise a wide variety of architectures. The most popular ones are the multilayer perceptron (MLP), convolutional neural networks (CNN), recurrent neural network (RNN) and generative adversarial networks (GANs). We describe these in turn, although the reader should be aware that many more options are available [24].

**Figure 1:**
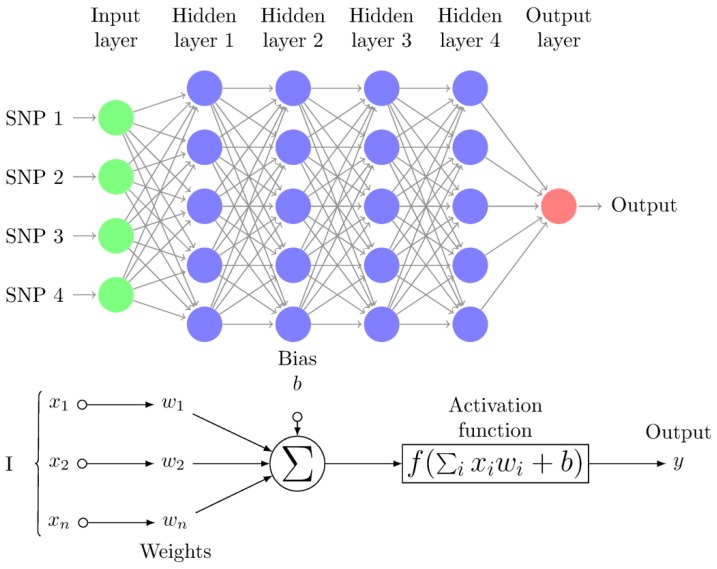
Multi-Layer Perceptron (MLP) diagram with four hidden layers and a collection of single nucleotide polymorphisms (SNPs) as input and illustrates a basic “neuron” with n inputs. One neuron is the result of applying the nonlinear transformations of linear combinations (x_i_, w_i_, and biases b). These figures were redrawn from tikz code in http://www.texample.net/tikz/examples/neural-network.

The multilayer perceptron network (MLP) is one of the most popular DL architectures, which consists of a series of fully connected layers called input, hidden, and output layers (Figure 1). In the context of genomic prediction, the first layer receives the SNP genotypes (x) as input and the first layer output is a weighted nonlinear function of each input plus the “bias” (i.e., a constant). The first layer output is then:

(2) $$z^{\left(1\right)}=b_{0}+W^{\left(0\right)}f^{\left(0\right)}\left(x\right)$$

where, x contains the genotypes of each individual, *b* is called the “bias” and is estimated together with the rest of weights W^(0)^, and *f* is a nonlinear function (available activation functions in Keras are in https://keras.io/activations/). In successive layers, the same expression as above is used except that neuron inputs of a given layer are the outputs from the previous layer ($z^{\left(k-1\right)}$):

(3) $$z^{\left(k\right)}=b_{k-1}+W^{\left(k-1\right)}f^{\left(k-1\right)}\left(z^{\left(k-1\right)}\right)$$

The final layer produces a vector of numbers if the target is a real-valued phenotype, or an array with probabilities for each level if the target is a class (i.e., a classification problem). Although MLPs represent a powerful technique to deal with classification or regression problems, they are not the best option to manage spatial or temporal datasets. To face these issues, other DL techniques such as convolutional neural networks, recurrent neural networks or deep generative networks have been proposed in recent years.

**Figure 2:**
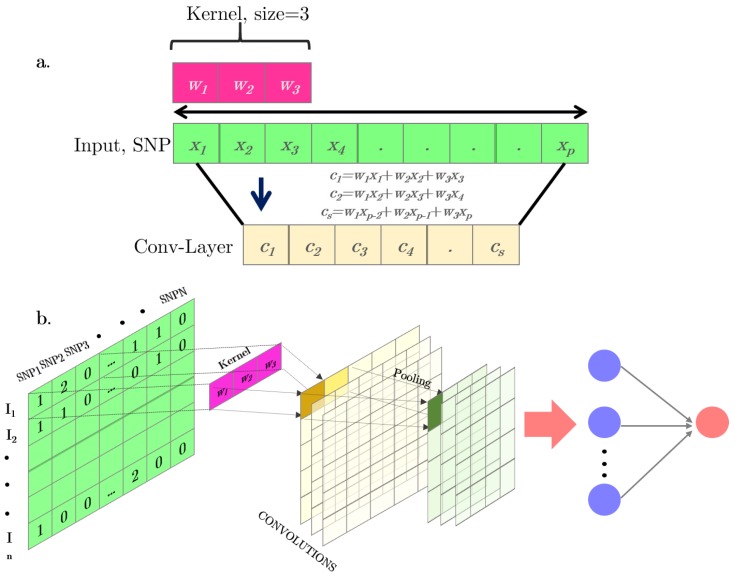
(**a**) Simple scheme of a one-dimension (1D) convolutional operation. (**b**) Full representation of a 1D convolutional neural network for a SNP-matrix. The convolution outputs are represented in yellow. Pooling layers after convolutional operations combining the output of the previous layer at certain locations into a single neuron are represented in green. The final output is a standard MLP.

Convolutional neural networks (CNNs) were proposed to accommodate situations where input variables are distributed along a space pattern, such as one-dimension (e.g., SNPs or text), and two- or three-dimensions (e.g., images). A CNNs is a special case of neural networks which uses convolution instead of a full matrix multiplication in the hidden layers [24]. A typical CNN is made up of dense, fully connected layers and “convolutional layers” (Figure 2b). In each convolutional layer, a convolutional operation is performed along the input of predefined width and strides. Each of these convolutional operations is called a “kernel” or a “filter” and is somewhat equivalent to a “neuron” in an MLP. An activation function is applied after each convolution to produce the output. Finally, an operation called “pooling” is usually applied to smooth out the result. It consists of merging the kernel outputs of different successive positions by taking the mean, maximum, or minimum of all values of those positions. One of the main advantages of convolutional networks is their capability to reduce the number of parameters to be estimated. These networks also have sparse interactions and are equivariant to translations. An illustration of a one-dimension (1D) convolution with a 3-K kernel size is depicted in Figure 2a. Figure 2b shows the steps involved in a convolutional network.

**Figure 3:**
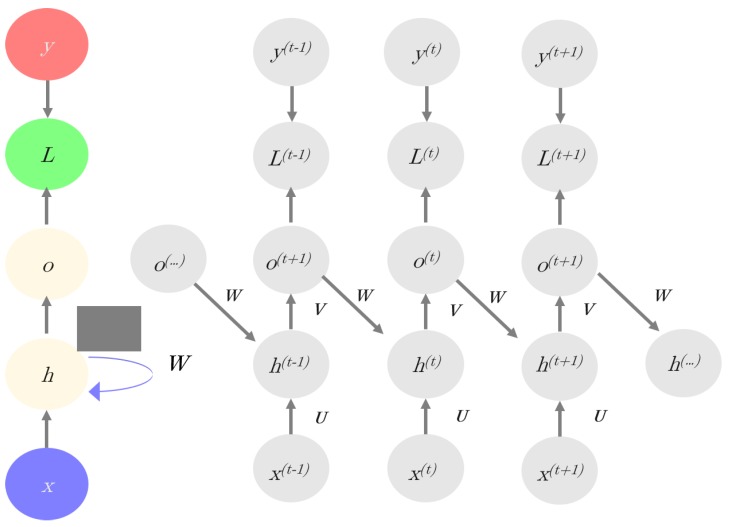
Scheme of recurrent neural networks (RNNs): The left part of the image (in colors) shows the whole network structure; whereas, the recursive structure of the network is shown in the right, where ***x*** represents inputs, *h* are the hidden layers, *o* = $Vh^{\left(t\right)}+b$ are the outputs (Equation (4b)), *y* are the target variables, and L is the loss function.

Recurrent neural networks (RNN) are specifically designed to model space-temporal structures because RNNs consider information from multiple previous layers [24,25]. In the RNN model, the current hidden layer is a nonlinear function of both the previous layer(s) and the current input (x). The model has memory since it has a bias based on the “past”. Figure 3 illustrates the RNN principles. Mathematically, an RNN is defined as follow:

(4a) $$h^{\left(t\right)}=f\left(Wh^{\left(t-1\right)}+Ux^{\left(t\right)}+b_{0}\right)$$

(4b) $${\hat{y}}^{\left(t\right)}=g\left(Vh^{\left(t\right)}+b_{1}\right)$$

Equation (4a) establishes that the current hidden layer *h^(^*^t)^ is a nonlinear function of the previous layer (*h*^(*t*−1)^) of the current input (*x*) and of bias, *b*_0_; *W* and *U* are weight matrices to be estimated. For instance, if *x* represents a sequence-like dataset, *x*^(*t*)^ refers to the value of *x* at time *t* (here we use time in a generalized way, to mean structure on the dataset). Equation (4b) indicates that the predicted output ${\hat{y}}^{\left(t\right)}$ is a nonlinear function of *h^(^*^t)^ and bias *b*_1_, where V is a weight matrix.

As in CNNs, RNNs share parameters, but in a slightly different manner. While CNNs share parameters using the convolutional kernel, RNNs do so through a recursive computational graph [24]. These two classes of networks are generally employed in different applications. RNNs are appropriate for recursive data, whereas, CNNs excel in image classification. The long short-term memory networks (LSTMs) [26] are a special kind of RNN designed to learn long-term dependencies and are the most popular RNN architecture. These networks are well known for their favorable convergence properties [27]. To our knowledge, RNNs have not been employed in genomic prediction, although Pouladi et al. [28] proposed a deep RNN combined with matrix factorization for genotype imputation and sequential phenotype prediction.

**Figure 4:**
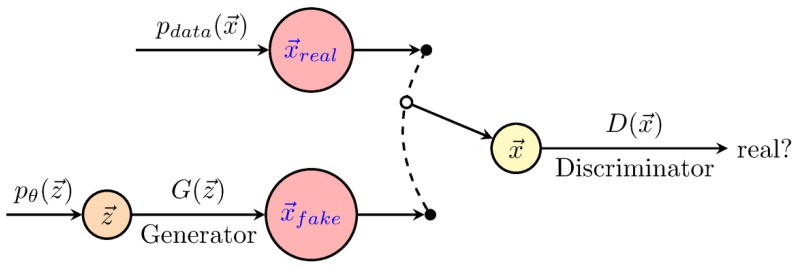
Scheme of generative adversarial networks (GANs). The generator (G), defines a probability distribution based on the information from the samples, whereas, the discriminator (D) distinguishes data produced by G from the real data. The figure was redrawn using code from http://www.texample.net/tikz/examples/neural-network.

The aim of generative models (Figure 4) is to infer a function to describe the structure of a dataset, i.e., as in the traditional generative-statistical models [29]. These techniques, in principle, model the joint distribution of the data, whatever its complexity. Among these methods, we briefly describe Boltzmann machines (BM) and generative adversarial networks (GANs). Boltzmann machines were proposed in 1983 by Hinton and Sejnowski [30], however, they have only become popular recently for the unsupervised pre-training of neural networks [31,32]. These networks aim to model complex joint probability distributions using Markov chain Monte Carlo and the maximum likelihood estimation (MCMC-MLE), which is computationally expensive. Generative adversarial networks [33] emerged to overcome this limitation. They are based on a simple but a powerful idea which involves training two networks simultaneously, i.e., the generator (G), which defines a probability distribution based on the information from the samples, and the discriminator (D), which distinguishes data produced by G from the real data. This scheme can be formalized as a minimax game theory rather than as an optimization problem. The model does not require an MCMC, but only requires a standard gradient descent in the optimization step, improving its computational performance. In a prediction context, GAN architectures can be used together with CNNs, MLPs or RNNs.

### 3.2. Algorithms and Optimization Issues

Irrespective of the architecture chosen, all DL algorithms are based on a few principles that are used to minimize the cost function and, hopefully, maximize the predictive ability. Here, we describe the main concepts.

Backpropagation and stochastic gradient descent are the bases for the modern revival of neuron-associated methods. Backpropagation [34] is a clever method which propagates the error backward at the output layer level. Then, the gradient of previous layers can be computed easily using the chain rule for derivatives, which greatly simplifies optimization in complex models. The basis of gradient descent [35] is also simple. The algorithm requires a set of initial solutions and a loss function, which usually has good mathematical behavior, i.e., it is a convex function or at least a quasi-convex function (metaphorically, this means reaching the lowest elevation point simply by going downhill). 

**Algorithm 1:** Stochastic Gradient Descent (SGD)Given the loss function *l*,**Input:** Training sample (*x_(train)_, y_(train)_*), regularization parameters Ω(θ), learning rate (α) and λ,  a penalization parameter. Initialize θ**Output:** Model parameters $\hat{\theta }=\left(b,W\right)$Initialize $\theta =\left\{W^{\left(1\right)},b^{\left(1\right)},\ldots ,W^{\left(L+1\right)},b^{\left(L+1\right)}\right\};$

**repeat**
 $\mathrm{for}\;i\;\epsilon \;n_{epochs\;}\mathrm{do}$  Given a training set (*x_(train)_, y_(train)_*),  Compute: $\Delta =-\frac{d}{d\theta }l(f\left(\left(x_{\left(train\right)};\theta \right),\;y_{\left(train\right)}\right)-\lambda \frac{d}{d\theta }l\left(\Omega \left(\theta \right)\right)$  $\theta_{i}\leftarrow \theta_{i-1}$
*+*
αΔ **end****until** stopping criteria/convergence;**where**$\frac{d}{d\theta }l(f\left(\left(x_{\left(train\right)};\theta \right),\;y_{\left(train\right)}\right)$ is the function gradient

Stochastic gradient descent (SGD, Algorithm 1) is one of the most widely used optimizers in DL. SGD, and a plethora of related methods, randomly partitions the whole dataset into subsets called “batches” or “minibatches” and updates the gradient using only that subset. The next batch is used in the next iteration. This intelligent strategy allows us, not only, to manipulate datasets of any arbitrary large size but also introduces stochastic noise that reduces the risk of converging at the local maxima. An “epoch” is the set of iterations that comprises all samples in the dataset. For the next epoch, a different data partition is used in each batch. In addition to batch size, SGD requires initial values for all parameters and specifies the “learning rate”, i.e., the value that controls the update of the gradient (Algorithm 1).

Note that Algorithm 1 incorporates a regularization term, Ω(θ) regularization consists of adding a “penalty” or “constraints” to the model parameters and incorporating a restriction over the weight (W) estimations in the loss function. The two most frequent regularizations are the L1 and L2 norms, which set restrictions on the sum of absolute values of W (L1) or of the square values (L2). A description of all the Keras implementation to DL optimization is found in https://keras.io/optimizers/.

Initial weight value is an additional factor that needs to be considered seriously and has deserved numerous contributions. Goodfellow et al. stated [24] (p. 293): “Our understanding of how the initial point affects generalization is especially primitive, offering little to no guidance for how to select the initial points." This is due to the fact that DL algorithms are iterative and the function to minimize cost is too complex. Therefore, initial values may affect whether convergence is attained or not. In our experience, it is critical to compare prediction performances for several different training runs with exactly the same hyperparameter values, using either random uniform or normal values. This should indicate the reliability of the initialization strategy.

The activation function is the mathematical function that transforms the linear input of the neuron into its output (Figure 1). The most popular activation function in the past was the logistic (sigmoid) function. This function often results in numbers that are either zeros or ones and is not flexible enough for most applications. Therefore, other functions are currently more popular, including “relu” or “selu”. Plots and descriptions of the most popular functions are found (e.g., in https://en.wikipedia.org/wiki/Activation_function) and are not further described here. Our recommendation is that the activation function should be considered as a hyperparameter to be optimized among two to four possible values, e.g., “tanh”, “relu”, “selu”, etc. In general, hidden units may need different activation functions from those of input or output layers. For instance, for classification problems, a “softmax” activation function in the output layer is frequently used. The table in the accompanying GitHub (https://github.com/miguelperezenciso/DLpipeline#loss) shows the most common combinations of loss function and last layer activation for different problems.

In a genomic prediction context for a quantitative trait, the simplest activation function is “linear”, which models only additive effects since it sums up through the allelic frequency of the SNP (zero, one, and two in a diploid organism). SNP are not numerical, but categorical data, although DL techniques only accept numerical input. A set of encoding methods was developed to overcome these constraints [36]. One hot encoding, which is simply recoding the three SNP genotypes as three 0/1 dummy variables, is the most popular approach for genomic prediction purposes. Using this approach, nonlinear relationships can be modeled by using nonlinear activation functions at the first layer [37].

### 3.3. Avoiding Overfitting

A model overfits when it cannot separate noise from signal. An overfitted model results from a poor fit on the validation set, i.e., prediction of unobserved data is very poor. This is one of the most, if not the most critical problem in DL applications. Most of the time in DL, optimization will be spent in avoiding an overfit of the data, in order to improve the predictive abilities of the algorithm. Given the unknown behavior of various algorithms, no general guidance is given, and trial-and-error is needed. Success here is very much dependent on the specific problem. There are broadly three non-mutually exclusive techniques to minimize the risk of overfitting: early stopping, regularization, and dropout.

Early stopping is the simplest and also, sometimes, the most effective strategy, since an excessive number of epochs tend to result in overfitting. Conversely, the number of epochs should be sufficient, as too few iterations result in underfitting. As with any hyperparameter optimization, the optimum number of epochs should be chosen using only the training set. This training subset is normally partitioned in a proper training dataset and a test subset which is used for internal cross-validation.

Regularization, as mentioned, is the procedure whereby constraints are imposed upon the weights’ estimates, which are incorporated in the general loss function (see SGD Algorithm 1). Regularization is a key parameter to avoid overfitting and should be very carefully optimized in your own data, perhaps using a grid or random search. In our experience with a large human dataset, the optimum regularization was surprisingly small [38]. This is likely a consequence of the algorithm failing to find the “regularities” in the data, which is needed for an optimum prediction of new data.

Dropout is another clever strategy that is specific to neural networks. Given an initial, completely connected network, dropout consists of setting to zero the output of a random subset of neurons. This strategy is equivalent to “bagging” (i.e., sampling) subnetworks to produce an ensemble (i.e., joint) estimator. The usual recommended dropout rate, i.e., percentage of inactivated neurons, is 20% to 50% [23]. However, in our experience with the UK Biobank human dataset, the optimized dropout rate was very small (<0.05%). Again, the optimum rate is a problem-specific issue and should be optimized with the data at hand. In practice, it is probably unnecessary to combine regularization, either L1 or L2 and dropout, whereas, early stopping is always a good practice.

## 4. Deep Learning Practicalities

### 4.1. Implementing Deep Learning

Implementing DL, despite all its theoretical and computational complexities mentioned above, is rather easy. This is thanks to Keras API [39] and TensorFlow [40], which allow all intricacies to be encapsulated through very simple statements. TensorFlow is a machine-learning library developed by Google. In addition, the machine-learning python library Sci-Kit Learn [41,42] is highly useful. Directly implementing DL in TensorFlow requires some knowledge of DL algorithms, and understanding the philosophy behind tensor (i.e., n-dimensional object) manipulations. Fortunately, this is avoided using Keras, a high-level python interface for TensorFlow and other DL libraries. Both TensorFlow and Keras are open source and freely available. Although alternatives to TensorFlow and Keras exist, we believe these two tools combined are currently the best option because they are simple to use and are well documented.

An analysis pipeline in Keras requires the following five main steps:

1. A model is instantiated. The most usual model is “sequential”, which allows adding layers with different properties step by step.
2. The architecture is defined. Here, each layer and its properties are defined. For each layer, the number of neurons, activation functions, regularization, and initialization methods are specified.
3. The model is compiled. The optimizer algorithm with associated parameters (e.g., learning rate) and loss function are specified. This step allows us to symbolically define the operations (i.e., graphs) to be performed later with actual numbers.
4. Training occurs. The model is fitted to the data and parameters are estimated. The number of iterations (i.e., epochs) and batch size are specified. The input and target variables need to be provided. The input data size must match that defined in step 2.
5. Model predictions are validated via cross-validation.

### 4.2. Training and Validating Deep Learning Architectures

The accompanying GitHub website (https://github.com/miguelperezenciso/DLpipeline) illustrates the principles described here. Together with implementation details, it contains a worked example that is comprised of data inspection, SNP filtering, and a comparison between penalized linear models and several DL architectures. As an example, we used the CIMMYT wheat public data described in BGLR software [28], which consists of 599 historical wheat lines that were genotyped with 1279 markers. The GitHub code can be easily modified to suit specific user needs.

One of the main difficulties when implementing DL models is to find the best hyperparameter configuration (the table in GitHub https://github.com/miguelperezenciso/DLpipeline#hyperparameter-optimization lists the main DL hyperparameters and their roles). This step requires some basic understanding of what is going on and a general idea of which hyperparameters to optimize, together with a plausible range of values. There are numerous tools to assist in this task, e.g., hyperas (https://github.com/maxpumperla/hyperas), keras_auto (https://github.com/Tony607/Keras_auto), genetic algorithms (for example https://github.com/paubellot/DL-Biobank), among others. Here, we used Talos (https://autonomio.github.io/docs_talos/). Talos allows grid, random, and probabilistic hyperparameter searches. A grid search is useful to systematically visualize the effect of a few predetermined hyperparameters and is recommended for final tuning. For real-world analyses, random or probabilistic searches is recommended [23]. For instance, it is probably unnecessary to go beyond three to five layers or over, for example, 100 neurons per layer. Testing up to four activation functions should probably capture all expected patterns. Similarly, each dropout, L1 or L2 regularization, does the same job, and therefore only one regularization hyperparameter is explored.

**Figure 5:**
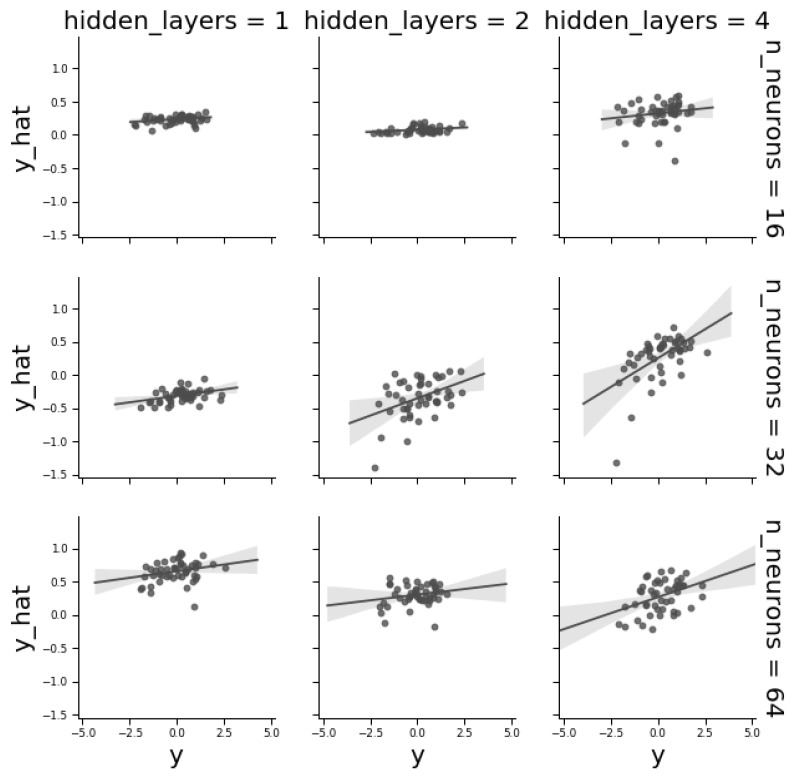
Correlations between observed and predicted phenotypes in the validation dataset as a function of the number of layers and of number of neurons per layer for the CYMMIT wheat data. Each dot corresponds to a single phenotype in the validation dataset. The best combination was 32 neurons and four hidden layers (correlation = 0.55).

To illustrate hyperparameter optimization, we studied the effect of network architecture by applying a “toy” grid search to a fully connected MLP architecture. An MLP is mainly defined by the number of layers and neurons per layer. Figure 5 plots observed vs. predicted ‘Centro Internacional de Mejoramiento de Maíz y Trigo’ (CIMMYT) phenotypes and shows that MLP architecture plays a critical role on predictive ability. Increasing the number of layers increased performance. Nevertheless, for a given number of layers, the number of neurons was not as critical, recalling that network tuning depends on the problem complexity and a good balance is needed between complexity and parsimony, since an increase in the number of neurons or layers may generate overfitting. In a classification context, extending the number of layers helps when classes are not linearly separable. In contrast, in a regression context, performance is mainly dependent on the number of variables, i.e., SNPs in this context [23].

To extend the results in Figure 5, where only the number of layers of neurons were varied, we simultaneously investigated the effect of dropout rate (0, 0.05%, 0.25%, or 0.5%), activation function (rectified linear units “relu” and sigmoid), number of neurons in first layer (eight, 64, 32, 24, or 128), number of hidden layers (one, two, four, or 10), kernel initialization (uniform or normal), and optimizer (Adam, Nadam, or SGD). The best combination that was found used a random search with Talos, included four hidden layers (64 neurons on the first layer), a mild dropout rate (0.05%), relu activation, Nadam optimizer, and normal kernel initialization. Overall, average performance increased with the number of neurons on the first layer, a dropout higher than zero, and using relu as the activation function. Note that the performance of the sigmoid function was poor because this activation function is not suited for regression-like problems.

**Figure 6:**
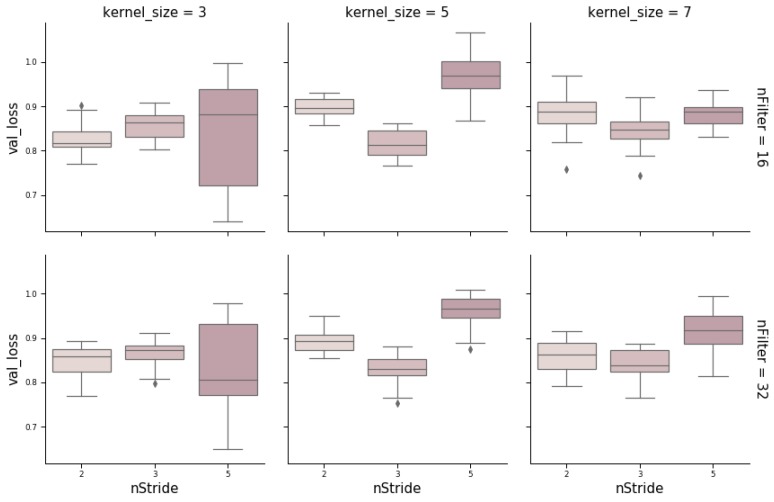
Box-plot representing loss values on the validation set with a convolutional neural network (CNN) architecture using different kernel sizes (3, 5, and 7), strides (1, 2, and 5), and number of filters (16 and 32).

**Figure 7:**
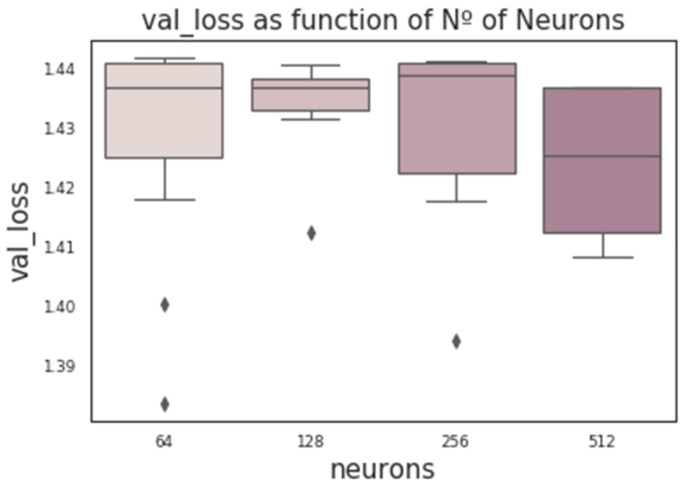
Box-plot representing the loss in the validation set vs. the number of neurons using RNN architecture.

CNNs contain additional hyperparameters to those of MLPs, such as kernel size (number of SNPs per window), stride, or number of filters (the number of output filters in the convolution). Figure 6 shows the influence of these hyperparameters obtained from a random grid search where other relevant hyperparameters were also tested (e.g., number of layers, neurons, kernel initializer, activation function, and dropout rate). In the wheat dataset, the optimum stride was the shortest (two SNPs) when kernel size was three, and a stride equal to three when kernel size ranged between five to seven SNPs. The number of filters seems not to have a large influence. Finally, we found that RNN performance was worse than the performance of MLPs or CNNs. Nevertheless, RNN performance improved (on average) with the number of neurons and when dropout was included (Figure 7). As with CNNs, the best performance was obtained using relu as the activation function and Nadam as the optimizer.

To summarize, even though hyperparameter is highly dependent on the specific context, some practical recommendations are made as follows: Nadam optimizer is preferred, use dropout (5–10% seems reasonable), use more than one hidden layer, and use relu as the activation function for regression problems. When using CNNs in a genomic prediction context, disequilibrium is likely to influence the optimum window size. The lower the disequilibrium, the shorter the optimum window size should be. In the CYMMIT data, optimum window size was small (~five SNPs). We found similar results for human data [38].

### 4.3. Feature Selection

Feature selection is an important step in the development of many machine learning prediction models, and has been widely applied in prediction problems e.g., [41,42]. Feature selection has several advantages, since it reduces overfitting and training time, as well as improves accuracy and has a long history in machine learning and in statistics [43,44,45]. For instance, the distorting effects of collinearity between highly correlated explanatory variables in a regression model are a well-known phenomenon. In principle, multicollinearity is not an important problem with DL or penalized methods such as LASSO or ridge regression, however, up to a point. In our experience, very large genomic datasets, such as over 100k SNPs, cannot be effectively handled due to the enormous number of parameters to be estimated. As a result, some feature (SNP) pre-selection is needed before feeding the DL algorithm.

In practice, we have found that SNP selection is needed for large-scale genomic prediction with DL [38]. In our experience, the simplest and most effective strategy among those tested is to select SNPs based on their individual *p*-value. This criterion is the one we recommend right now. Setting a constraint on the list of SNPs ranked by *p*-value, such as a minimum distance between selected SNPs or a minimum allele frequency did not help and was even detrimental. Randomly choosing SNPs was by far the worst strategy and is not recommended. It is argued that individual *p*-values may not capture complex gene relationships such as epistasis. However, individual additive effects capture a fraction of epistasis and are, therefore, indirectly including genes with nonadditive behaviors. The behavior of CNNs is interesting with regard to SNP selection. Since CNNs exploit the spatial correlation between input variables, we hypothesized that equal SNP spacing might ameliorate CNN performance as compared to choosing SNPs by absolute *p*-value. However, the contrary was observed by Bellot et al. [38] due to the “granularity” of the SNP *p*-value distribution along the genome; there were hotspots near strong candidate genes together with deserts in large genomic regions. We believe that the optimum SNP choice for prediction is an open research area in DL. In the specific case of DL, other pre-selection criteria such as LASSO (L1 regularization) could be explored.

### 4.4. Interpreting DL

In a very broad sense, model interpretation refers to extracting relevant information from a dataset analysis. Loosely speaking, the interpretation aims to not produce black box predictive models. While it is true that interpretability might not be relevant in an exclusive prediction problem, e.g., to obtain the best genotypes and accelerate selection progress in an animal or plant breeding scheme, it can be a hindrance in prediction for medical purposes. For instance, in a healthcare context, it is not only necessary to predict, but also to understand the “reasons” behind predictions in order to take informed decisions about patients’ treatment [46].

Although DL architectures are not designed for inference, i.e., hypothesis testing purposes, approaches exist to interpret DL results a posteriori [13]. Here, we focus on one of the most important aspects of interpretation, the extraction of feature importance. Several indirect metrics have been proposed in this area. For instance, Sheehan and Song [47] used two methods to investigate the most relevant input variables. In the permutation approach, they permuted the values of a given input variable across samples and measured the decrease in prediction accuracy. In the perturbation method, they added a small signal error to the variable and compared predictions with new data. A major drawback of these approaches is that they are computationally expensive, and therefore can only be applied to a small number of variables, and that they cannot ascertain interactions between variables.

Eraslan et al. [13] also discussed model interpretability in terms of “feature importance” scores and list several methods to compute them. Similarly, Bellot et al. (unpublished analysis on the UK Biobank) computed the SNP z-score of the first neuron layer as an approximate metric of SNP relevance in MLP. The correlations between SNP solutions for LASSO or ridge regression and the SNP z-scores were approximately one and 0.90 when using 10k and 50k SNPs, respectively. The performance of all methods was similar. Since the weights given to the same SNP were similar in both linear methods and in DL, some aspects of DL can be interpreted as in penalized linear methods, at least for shallow networks.

Schwab et al. [48] presented an approach to estimate feature importance based on a “mixture of experts” neural network. The idea behind this architecture was to split the input space into small clusters, model each part separately, and then combine all partial models to obtain the global solution. Data was processed at either observation or variable levels. When the last approach was used, weights for each variable were estimated. The authors applied their proposal to a multi-carcinoma gene-expression dataset and obtained a higher accuracy in comparison with other established methods without a loss in computational speed.

Dhurandar el at. [49] considered a completely different approach. The goal was to transfer information from a high performance, uninterpretable DL to a simpler and less performing model, but easier to interpret. The rationale was that these simpler models actually give insight into the underlying biological processes. To our knowledge, this method has not been applied to genomic prediction. Reichstein et al. [50] advocated for the use of hybrid systems (DL and conventional modeling) to improve geophysical modeling and predictions. How these ideas are actually implemented in genomic prediction, however, remains to be investigated.

## 5. Applications of Deep Learning to Genomic Prediction

The number of reports on DL applications is small and those we found thus far are listed in Table 2. To our knowledge, the first application of DL for genomic prediction was the MSc thesis by Mcdowell [51]. He compared prediction performance of several MLP architectures, differing in the regularization approach (i.e., dropout and weight decay), with penalized methods LASSO, ridge regression, and elastic net. In relatively small datasets of cereals and Arabidopsis with a small number of markers, he found that MLPs outperformed penalized methods only if regularization was applied and then only in 50% of the analyses and for a modest margin, ~3%.

Further studies have also employed MLPs [52,53,54]. In [52], the authors compared MLP with GBLUP, equivalent to ridge regression, using cereal data [18]. They reported that GBLUP was consistently better than MLPs when genotype by environment interaction (GxE) was included in the model, whereas, the opposite occurred when data were not corrected for GxE effects. The same authors generalize these analyses to multiple trait prediction and ordinal traits, with similar conclusions [52]. In this sense, it is worth noting that DL methods are able to learn better than parameterized models when the model is not correct. We believe DL-based genomic prediction can be of particular interest in these scenarios. Very often in plants, the whole genetic value (including nonadditive effects) is of interest, since the genotype can be reproduced vegetatively and predicting only the additive effects is insufficient.

In [37], the author implements an “approximate Bayesian neural network” architecture to predict complex traits in a simulated and real pig dataset. This approach avoids overfitting through a combination of dropout and weight decay procedure. A positive outcome of this study is not only that DL has a better performance in comparison to classical models, but also that the importance of variables (SNPs) can be quantified when a linear activation function is used.

Recently, the group of Liu and Wang [55], compared a deep MLP (21 layers) with LASSO and regression trees in the 2018 Syngenta Crop Challenge dataset. The target was the prediction of hybrid corn performance across locations. In this case, MLP was by far the best method, followed by regression trees. It is not clear why linear methods (LASSO in this case) performed much worse than MLPs. Perhaps, as conjectured above, because nonadditive effects in maize hybrids are very strong.

Overall, CNNs have shown better performance than classical MLPs for genomic prediction. This could be explained by the fact that CNNs are able to exploit part of the correlation between nearby SNP positions (i.e., disequilibrium). In the most comprehensive and large-scale study so far [38], we found that CNNs could be competitive to penalized linear methods in the human UK Biobank data, depending on the phenotype. In that study, all methods performed similarly for height, a highly heritable phenotype, although CNNs were slightly, but consistently worse. For other traits, the performance of some CNNs was comparable or slightly better than linear methods. Interestingly, it was found that the performance of MLPs was highly dependent on the SNP set and phenotype, whereas, that was not the case for penalized linear methods. This emphasizes the need to carefully optimize hyperparameters and DL architecture. For the traits evaluated in the UK Biobank study, CNN performance was competitive with linear models, but DL did not outperform linear models by a sizable margin. Along this vein, the group of Liu and Wang [55] claimed that CNNs resulted in better predictions than Bayes A or ridge regression in soy bean data, but no details were given. Furthermore, the study lead by Ma [57], compared CNNs, MLPs, and GBLUP and reported a slightly better performance for CNNs than GBLUP, which were more accurate than MLPs. This study investigated 2000 wheat lines and ca. 33k markers.

The evidence in recent literature indicates that some DL methods can be competitive with linear methods, but not in all cases. Notably, genomic prediction may not be the best scenario for DL because the actual target (breeding value) is unknown and only a noisy variant thereof. Furthermore, features (SNPs) have highly leptokurtic distributions, due to the large number of rare SNPs which can be highly redundant due to linkage disequilibrium. Except in the case of Bellot et al. [38], DL has not been applied in large datasets. In their case, it was necessary to preselect 10–50k SNPs out of the 500k initially available. In [52,53], the authors do not cite any SNP preselection procedure, although their sample size was much smaller than in [38]. Among the DL methods, CNNs seem to be the most promising ones, and we suggest that a potential area of research is to optimize CNN architecture for the specific purpose of genomic prediction, much as there are specialized architectures for image analysis.

## 6. Perspectives and Conclusion

Deep learning actually comprises a wide variety of algorithms that depend on numerous hyperparameters. Here we have provided the main theoretical foundations and parameters composing the architecture of a deep network. To facilitate its use, we also provide Keras scripts that should allow a painless introduction to the unexperienced user (https://github.com/miguelperezenciso/DLpipeline). Beware that DL is not a plug-and-play tool; utmost care should be employed with hyperparameter tuning, recalling that each new dataset will require fine tuning. DL theory is still immature, and most applications are heavily based on heuristics.

Despite potential advantages, DL presents some difficulties in its implementation. One of the main difficulties is that DL algorithms are prone to overfitting, i.e., they can perform well in training and poorly in testing, and can be difficult to optimize [58,59]. In addition, DL algorithms depend on numerous hyperparameters and finding the optimum value can be challenging. Furthermore, DL needs very large datasets for training [23,59], and these may not be available in all genomic prediction settings. This can be a serious challenge for many genomic applications, since datasets tend to be highly heterogeneous and of small size. In these cases, one solution is “transfer learning”, which is to re-use models that have been trained with similar datasets. The interested reader is referred to [13] and references therein. In the case of genomic prediction, the most straightforward manner of transfer learning would be to use prior information from previous genome-wide association analysis of similar phenotypes. For instance, the Animal QTL database (https://www.animalgenome.org/cgi-bin/QTLdb/index) lists published genome regions associated with numerous traits of interest in livestock. The DL algorithms would need to be modified such that a larger prior weight is given to SNPs residing in candidate genes or regions. To our knowledge, this approach has not been envisaged with DL, although similar ideas have been applied in standard genomic prediction methods (e.g., [60]).

Another limitation of the DL algorithms is the interpretability of their results. A prediction is given by the algorithm but, generally, its relation to the input variables cannot be ascertained in a straightforward manner. For strict genomic prediction purposes, this may not be a serious issue, but it is a deterrent for many other applications, e.g., we are usually interested in knowing the causal genes when predicting disease liability.

So far, all applications of DL to genomic prediction have been done with “standard” quantitative or binary phenotypes. Although limited, evidence so far indicates that we should not expect dramatic improvements with DL in this field. However, DL tools are especially suited for predicting whole genetic value, as suggested by S. Gezán (pers. comm.). Furthermore, with current automatic phenotyping technologies, numerous non-standard phenotypes can be recorded such as images, videos, and even sound. Deep learning, and CNNs in particular, appear as the most promising predictive tool with these kinds of phenotypes. This could be due in part to the fact that convolutional filters may capture some functional sequence motifs.

## 7. Practical Recommendations

1. Before starting, inspect the data, both SNPs and phenotypic distributions. Look for unexpected, weird patterns that may cause biases. Standardize the variables and targets.
2. Use Keras with TensorFlow, together with Sci-Kit Learn, a collection of well documented, easy-to-use machine learning modules. Reuse, but test, available public software whenever possible.
3. Exercise prudence if extremely good or poor results are obtained. Ample literature does support that differences between methods should not be dramatic.
4. Do not be too ambitious. Is your data set big enough to fit such complex models?
5. Dedicate enough time and thinking to optimize hyperparameters. Finely tune early, stopping to improve prediction performance. If the number of SNPs is too large, preselect different subsets according to the *p*-value or try other criteria.
6. Once an optimum hyperparameter set has been decided, restart the algorithm several times to assess the influence of initial values.

## Figures and Tables

**Table 1:** Some usual terms in deep learning (DL) methodology.

Term	Definition
Activation function	The mathematical function f that produces neuron’s output f(**w’x** + b).
Backpropagation	Backpropagation is an efficient algorithm to compute the loss, it propagates the error at the output layer level backward.
Batch	In stochastic gradient Ddescent (SGD) algorithm, each of the sample partitions within a given epoch.
Convolution	Mathematically, a convolution is defined as an “integral transform” between two functions, where one of the functions must be a kernel. The discrete version of the operation is simply the weighting sum of several copies of the original function (*f*) shifting over the kernel.
Convolutional neural network	A CNN is a special case of neural networks which uses convolution instead a full matrix multiplication in the hidden layers. A typical CNN is made up of dense fully connected layers and “convolutional layers”.
Dropout	Dropout means that a given percentage of neurons output is set to zero. The percentage is kept constant, but the specific neurons are randomly sampled in every iteration. The goal of dropout is to avoid overfitting.
Early stopping	An anti-overfitting strategy that consists of stopping the algorithm before it converges.
Epoch	In SGD and related algorithms, an iteration comprising all batches in a given partition. In the next epoch, a different partition is employed.
Kernel = Filter = Tensor	In DL terminology, the kernel is a multidimensional array of weights.
Generative adversarial network (GAN)	GANs are based on a simple idea: train two networks simultaneously, the generator (G), which defines a probability distribution based on the information from the samples, and the discriminator (D), which distinguishes data produced by G from the real data.
Learning rate	Specify the speed of gradient update (α in Algorithm 1).
Loss	Loss function measures how differences between observed and predicted target variables are quantified.
Neuron	The basic unit of a DL algorithm. A “neuron” takes as input a list of variable values (**x**) multiplied by “weights” (**w**) and, as output, produces a non-linear transformation f(**w’x** + b) where f is the activation function and b is the bias. Both **w** and b need to be estimated for each neuron such that the loss is minimized across the whole set of neurons.
Neuron layer	“Neurons” are arranged in layers, i.e., groups of neurons that take the output of previous group of neurons as input (Figure 1).
Multilayer perceptron (MLP)	Multilayer perceptron network is one of the most popular NN architectures, which consists of a series of fully connected layers, called input, hidden, and output layers. The layers are connected by a directed graph.
Optimizer	An algorithm to find weights (***w*** and *b*) that minimize the loss function. Most DL optimizers are based on stochastic gradient descent (SGD).
Pooling	A pooling function substitutes the output of a network at a certain location with a summary statistic of the neighboring outputs. This is one of the crucial steps on the CNN architecture. The most common pooling operations are maximum, mean, and median.
Recurrent neural Network (RNN)	RNN architecture considers information from multiple previous layers. In an RNN, the current hidden layer is a nonlinear function of both the previous layer(s) and the current input (x). The model has memory since the bias term is based on the “past”. These networks can be used in temporal-like data structures.
Stochastic gradient descent (SGD)	An optimizing algorithm that consists of randomly partitioning the whole dataset into subsets called “batches” or “minibatches” and updates the gradient using only that data subset. The next batch is used in the next iteration.
Weight regularization	An excess of parameters (weights, **w**) may produce the phenomenon called “overfitting”, which means that the model adjusts to the observed data very well, but prediction of new unobserved data is very poor. To avoid this, weights are estimated subject to constraints, a strategy called “penalization” or “regularization”. The two most frequent regularizations are the L1 and L2 norms, which set restrictions on the sum of absolute values of ***w*** (L1) or of the square values (L2).

**Table 2:** Applications of deep learning to genomic prediction.

Study	Species	Approx. N	Approx. No. SNPs	Performance *
Mcdowell [51]	Arabidopsis, Maize, wheat	270–400	70–1k	MLP ≥ PL
Liu and Wang [55]	Soybean	5k	4k	CNN > RR-BLUP, Lasso-Bayes, Bayes A
Rachmatia et al. [56]	Maize	300	1k	PL > DBN
Bellot et al. [38]	Human	100k	10k–50k	PL ≥ CNN > MLP
Ma et al. [57]	Wheat	2k	33k	CNN ~ PL ~ GBLUP > MLP
Montesinos-López et al. [52]	Maize, wheat	250–2k	12k–160k	GBLUP > MLP
Montesinos-López et al. [53]	Wheat	800–4k	2k	GBLUP > MLP
Khaki and Wang [54]	Maize	2k genotypes (150k samples)	20k	DL > PL
Waldmann [37]	pig	3226 (simulated) 3534 (real)	10k, 50k	DL > GBLUP/BayesLasso

* PL, penalized linear method; DBN, deep belief network.

## Supplementary Materials

This manuscript is accompanied by a GitHub site https://github.com/miguelperezenciso/DLpipeline, which contains basic vocabulary and practical issues on DL, a jupyter notebook with an analysis pipeline, and example data.
